# Comparison of bedside abdominal ultrasonography and abdominal radiography in predicting surgical intervention in neonatal necrotising enterocolitis

**DOI:** 10.3389/fped.2026.1895567

**Published:** 2026-07-09

**Authors:** Hongwei Huang, Yang Yuan, Fanyue Qin, Huifang Dong, Xuanxuan Chen, Xiangyang Chu, Qi Liu, Hua Huang

**Affiliations:** 1 Department of Neonatal Surgery, The Third Affiliated Hospital of Zhengzhou University, Zhengzhou, China; 2 Department of Neonatology, The Third Affiliated Hospital of Zhengzhou University, Zhengzhou, China

**Keywords:** necrotising enterocolitis, prediction model, radiography, surgical indication, ultrasonography

## Abstract

**Objective:**

To compare bedside ultrasonography (US) and abdominal radiography (AXR) within the same cohort for their ability to discriminate the need for surgical intervention in neonatal necrotising enterocolitis (NEC), and to evaluate the value of combining the two modalities.

**Methods:**

A total of 509 neonates admitted to our neonatal intensive care unit from January 2015 to December 2023 were included; all had a clinical diagnosis of NEC and underwent abdominal radiography (a supine cot-side view in all infants, with an additional erect view obtained in the radiology department only when the infant was stable enough to be transported) and bedside US within 24 h. They were divided into a surgical group (*n* = 88) and a conservative group (*n* = 421) by clinical outcome. Eight signs from each of AXR and US were recorded as binary variables. After univariate screening by the *χ*^2^ or Fisher’s exact test, an AXR model, a US model, and a combined model were fitted by multivariable logistic regression. Model discrimination was assessed by the area under the receiver operating characteristic curve (AUC); AUCs were compared by the DeLong test, and their 95% confidence intervals (CIs) were estimated from 1,000 bootstrap resamples. The added value of the combined model was measured by the net reclassification index (NRI) and the integrated discrimination improvement (IDI). Calibration was assessed by the Hosmer–Lemeshow (HL) test, the Brier score, and calibration curves. Firth’s penalised logistic regression was used as a sensitivity analysis for signs with low cell counts. For neonates diagnosed with NEC during the same period but without US, the US signs were imputed by multiple imputation with chained equations (*m* = 20) and the models refitted, to assess the potential selection bias of the complete-case analysis.

**Results:**

The surgical group had a lower gestational age [33.0(30.0, 36.0) week] and birth weight [1,730(1,235, 2,205) g] than the conservative group (35.0[32.0, 36.0] week; 2,200[1,727.5, 2,732.5] g; both *P* < 0.001). On multivariable analysis, five signs were independent predictors of surgical intervention: AXR portal venous gas [odds ratio (OR) = 32.76, 95% CI 7.11–150.94], AXR pneumoperitoneum (OR = 11.91, 95% CI 1.81–78.58), US complex peritoneal effusion (OR = 16.94, 95% CI 3.65–78.76), US peritoneal effusion (OR = 7.53, 95% CI 4.04–14.03), and US portal venous gas (OR = 2.71, 95% CI 1.33–5.51). The AUCs of the AXR, US, and combined models were 0.696, 0.842, and 0.862. On the DeLong test, both the US and combined models were superior to the AXR model (both *P* < 0.001), whereas the difference between them was not significant (*P* = 0.067). The NRI and IDI of the combined model were 1.074 and 0.285 relative to the AXR model and 0.823 and 0.106 relative to the US model. All three models were well calibrated (HL test *P* > 0.05; Brier scores 0.116, 0.091, and 0.075).

**Conclusion:**

For neonates with a clinical diagnosis of NEC, bedside US was superior to AXR in the overall discrimination of the need for surgical intervention. AXR portal venous gas and pneumoperitoneum were complementary to the US findings of complex peritoneal effusion and peritoneal effusion; although adding AXR to US did not raise the AUC over US alone, it still added value in reclassifying borderline cases. US and AXR may serve as complementary modalities in the routine imaging work-up of NEC.

## 1 Introduction

Necrotising enterocolitis (NEC) is a common and serious gastrointestinal emergency of the neonatal period. Its incidence among preterm infants is approximately 7%–11%, and mortality remains high among infants who develop a severe, surgical phenotype (1, 2). The modified Bell staging system is still in use (3, 4), but it relies mainly on pneumoperitoneum, a relatively late sign, as the indication for surgery, which may delay intervention. In its 2025 evidence-based guideline, the European Reference Network for Inherited and Congenital Anomalies (ERNICA) advised against treating any single radiographic sign, such as a fixed bowel loop, as an absolute indication for surgery, recommending instead that imaging be interpreted together with the overall clinical picture (5); this reflects a broader shift in NEC assessment from a single sign towards the combined evaluation of multiple findings. Deciding when to operate in NEC remains a shared challenge for neonatal surgeons and neonatologists.

Abdominal radiography (AXR) is the main imaging method for NEC. Portal venous gas, pneumatosis intestinalis, and pneumoperitoneum are fairly specific for perforation or bowel necrosis, but their sensitivity depends heavily on patient positioning, the distribution of bowel gas, and the reader's experience (3, 6, 7). Bedside ultrasonography, using a high-frequency probe, can track bowel-wall perfusion, bowel-wall thickness, and the character of free fluid in real time, and several centres have already adopted it as an adjunct in NEC assessment, including in extremely preterm infants at the threshold of viability, in whom radiographs are often non-specific (8–10). Direct comparisons of the two modalities within a single cohort, however, remain scarce, and real-world data from tertiary NICUs in China are even more limited. Using the same source cohort, our group has previously published a nomogram for the risk of surgical intervention that combined clinical, laboratory, and imaging variables (11); imaging carried substantial weight in that model, but the two imaging methods were not compared directly. The present study restricted the analysis to 509 neonates who had undergone both radiography and ultrasonography and applied univariate analysis, multivariable logistic regression, DeLong testing of AUCs, and the net reclassification index (NRI)/integrated discrimination improvement (IDI) reclassification metrics in turn, so as to evaluate the discriminative performance of each imaging method and of the two combined, and to provide single-centre evidence for a local NEC imaging pathway.

## 2 Materials and methods

### 2.1 Study population

This was a single-centre retrospective cohort study. We screened all neonates admitted to our neonatal intensive care unit (NICU) between January 2015 and December 2023 who met the clinical diagnosis of NEC at modified Bell stage IIa or above (3) and kept those who had undergone abdominal radiography and bedside ultrasonography within 24 h. Eligible neonates had a gestational age of 24–41 weeks and a postnatal age of ≤28 days, an interval of no more than 24 h between the first radiograph and the first ultrasound, and complete data on clinical outcome (surgery or conservative treatment). We excluded neonates with congenital gastrointestinal malformations (such as intestinal atresia or malrotation), severe congenital heart disease or chromosomal abnormalities, those whose families withdrew treatment or who were discharged against medical advice within 48 h of admission, and those with missing or substandard images. The final cohort comprised 509 neonates: 88 underwent surgery (surgical group) and 421 were treated conservatively and discharged after recovery (conservative group). Conservative (non-operative) management comprised nil-by-mouth bowel rest, gastric decompression, broad-spectrum intravenous antibiotics, parenteral nutrition, and supportive intensive care, without surgical intervention. The study was approved by the institutional medical ethics committee (approval number: 2025-395-01); informed consent was waived because of the retrospective design.

### 2.2 Data collection

Four categories of data were retrieved from the electronic medical record and the picture archiving and communication system (PACS). General information covered sex, gestational age, birth weight, mode of delivery, and feeding pattern. Clinical signs covered abdominal distension, worsening distension, bloody stool, vomiting, abdominal tenderness, abdominal guarding, abdominal mass, abdominal-wall erythema, and changes in bowel sounds. Laboratory data covered C-reactive protein (CRP), the complete blood count, procalcitonin, serum sodium, and blood culture. Imaging signs are described in Section 2.3.

### 2.3 Imaging assessment

Radiographs were obtained as a supine view at the cot side in the NICU for all infants; an additional erect (upright) view was acquired in the radiology department only in infants whose condition was stable enough to allow transport. Films were reported by an attending (associate-chief or more senior) radiologist with more than 10 years of experience in pediatric radiology, who recorded eight signs: intestinal gas, rigid bowel course, widened interloop spacing, fixed bowel loop, equivocal intramural gas, pneumatosis intestinalis, portal venous gas, and pneumoperitoneum. “Equivocal intramural gas” was defined as scattered punctate or linear lucencies seen on only a single plane or a single segment, insufficient for a definite diagnosis; “pneumatosis intestinalis” required continuous or clustered intramural gas appearing consistently on multiple planes or multiple segments. Bedside ultrasonography was performed at the cot side by the radiology/ultrasound department (an attending sonologist with more than 5 years of experience in neonatal ultrasound) and reported by a radiologist; it was therefore a department-performed, radiology-interpreted bedside study, distinct from provider-performed point-of-care ultrasound (POCUS). Scanning was performed with a high-frequency linear probe (8–14 MHz), supplemented by a convex probe, and covered four consecutive quadrants from the upper abdomen to the pelvis, followed suspicious segments along the colon, and examined the portal vein at the hepatic hilum and any free peritoneal fluid. Peristalsis was observed for at least 30 s in each segment, and bowel-wall perfusion was assessed with a low-velocity colour-Doppler preset. Eight ultrasound signs were likewise recorded: intestinal gas, bowel-wall thickening [>2.7 mm; the upper normal limit of 1.1–2.6 mm reported by Faingold et al. was used (12)], bowel-wall ischaemia (loss or marked reduction of the colour-Doppler signal), equivocal intramural gas, pneumatosis intestinalis, portal venous gas, peritoneal effusion (any free fluid), and complex peritoneal effusion (turbid echoes, septation or loculation superimposed on the effusion). All imaging signs were entered as binary variables (present/absent) on the basis of the original radiology and ultrasound reports and the images stored in the PACS. Because these reports were issued before any decision on surgery as part of routine clinical care, the reading radiologists were blinded to the eventual surgical outcome by the temporal sequence of care; the images were not, however, re-read independently by a second observer, and therefore, inter-observer agreement (*κ*) could not be assessed (see the Limitations section).

### 2.4 Determination of surgical intervention

For most infants, the decision to operate was made jointly, at the cot side, by associate-chief or more senior physicians from neonatal surgery and neonatology who were members of the team caring for the infant, with reference to the modified Bell stage IIIB criteria and to rapid clinical deterioration (persistent metabolic acidosis, progressive thrombocytopenia, shock, worsening abdominal-wall erythema). Pneumoperitoneum was treated as an absolute indication: once it was identified on radiography and a benign cause (for example, air tracking from mechanical ventilation or from an extra-abdominal source) was excluded, the infant proceeded to immediate operation irrespective of the other criteria. Surgical procedures included laparotomy, bowel resection and anastomosis, enterostomy, and simple peritoneal drainage.

### 2.5 Statistical analysis

Analyses were run in Python 3.9. Descriptive statistics and logistic regression used pandas 2.0, statsmodels 0.14, and scikit-learn 1.3; the DeLong test, NRI/IDI, Firth’s penalised regression, and multiple imputation were handled by compare_auc_delong_xu, nricalc, firthlogist 0.5 and the IterativeImputer in sklearn, respectively. None of the continuous variables passed the Shapiro–Wilk test for normality, and therefore, they were reported as the median (first and third quartiles, Q_1_, Q_3_) and compared between groups by the Mann–Whitney U test. Categorical variables were given as *n* (%) and compared by the *χ*^2^ test or Fisher’s exact test, depending on cell counts. For modelling, imaging signs with a univariate *P* < 0.10 were first selected from the 509 cases, and three models were then fitted by multivariable logistic regression (one with radiographic signs only, one with ultrasound signs only, and one with both), each using surgery as the outcome. Because pneumoperitoneum and portal venous gas on radiography were infrequent in the conservative group and produced unduly wide 95% CIs, the models containing such signs were additionally fitted with Firth’s penalised logistic regression. Model performance was examined in three respects: discrimination by the area under the receiver operating characteristic curve (AUC), with between-model differences tested by DeLong's method and the 95% CIs of the AUC estimated from 1,000 bootstrap resamples; the added value of the combined model by the NRI and IDI; and calibration by the Hosmer–Lemeshow (HL) test, the Brier score, and calibration curves. The cut-off was set at the point of maximum Youden index, at which sensitivity, specificity, and positive and negative predictive values were reported. Because the complete-case analysis required both radiography and ultrasonography within 24 h and might introduce selection bias, neonates diagnosed with NEC during the same period but without ultrasonography were included, their ultrasound signs imputed by multiple imputation with chained equations (*m* = 20), and the models refitted as a sensitivity analysis. Reporting followed the TRIPOD statement. The 95% CIs of the AUC were derived from 1,000 bootstrap resamples of the development cohort; no separate (temporal or external) validation cohort was available, and therefore, only this single-cohort, bootstrap-based estimate of apparent performance is reported, and the absence of an independent validation cohort is acknowledged as a limitation. All tests were two-sided, with *P* < 0.05 considered statistically significant.

## 3 Results

### 3.1 Baseline characteristics

Of the 509 neonates, 272 were boys and 237 girls, with no difference in sex distribution between the groups (*χ*^2^ = 0.01, *P* = 0.911). The surgical group had a gestational age of 33.0 (30.0, 36.0) weeks and a birth weight of 1,730 (1,235, 2,205) g, against 35.0 (32.0, 36.0) weeks and 2,200 (1,727.5, 2,732.5) g in the conservative group; both differences reached *P* < 0.001 (Table 1).

**Table 1:** Baseline characteristics of the two groups.

Variable	Surgical (*n* = 88)	Conservative (*n* = 421)	Statistic	*P*
Male/female, *n* (%)	48 (54.5)/40 (45.5)	224 (53.2)/197 (46.8)	*χ*^2^ = 0.01	0.911
Gestational age, week, *M* (Q_1_,Q_3_)	33.0 (30.0, 36.0)	35.0 (32.0, 36.0)	*Z* = −4.21	<0.001
Birth weight, g, *M* (Q_1_,Q_3_)	1,730 (1,235, 2,205)	2,200 (1,727.5, 2,732.5)	*Z* = −5.95	<0.001

Continuous variables were compared by the Mann–Whitney *U* test and categorical variables by the *χ*^2^ test.

### 3.2 Clinical signs and laboratory findings

Five signs (worsening abdominal distension, abdominal tenderness, abdominal guarding, abdominal mass, and abdominal-wall erythema) were far more frequent in the surgical group (all *P* < 0.001). Among laboratory variables, CRP was markedly higher in the surgical group, whereas the white-cell count, absolute lymphocyte count, platelet count, and serum sodium were lower (all *P* < 0.05) (Table 2).

**Table 2:** Main clinical signs and laboratory findings of the two groups.

Variable	Surgical (*n* = 88)	Conservative (*n* = 421)	OR/*Z*	*P*
Worsening abdominal distension, *n* (%)	43 (48.9)	23 (5.5)	OR = 16.54	<0.001
Bloody stool, *n* (%)	45 (51.1)	299 (71.0)	OR = 0.43	<0.001
Abdominal tenderness, *n* (%)	54 (61.4)	18 (4.3)	OR = 35.56	<0.001
Abdominal guarding, *n* (%)	56 (63.6)	12 (2.9)	OR = 59.65	<0.001
Abdominal mass, *n* (%)	5 (5.7)	0 (0.0)	–	<0.001
Abdominal-wall erythema, *n* (%)	11 (12.5)	3 (0.7)	OR = 19.90	<0.001
CRP, mg/L, *M* (Q_1_,Q_3_)	71.01 (41.49, 110.47)	3.52 (0.88, 15.01)	*Z* = −13.74	<0.001
WBC, ×10^9^/L	6.05 (4.21, 10.68)	9.96 (7.99, 12.70)	*Z* = −7.11	<0.001
Lymphocytes, ×10^9^/L	1.50 (0.98, 2.40)	3.41 (2.48, 4.54)	*Z* = −9.08	<0.001
Platelets, ×10^9^/L	212.5 (116.75, 284.25)	271 (211, 355)	*Z* = −5.40	<0.001
Serum sodium, mmol/L	136.0 (134.0, 138.0)	138.0 (136.0, 140.0)	*Z* = −5.79	<0.001

CRP, C-reactive protein; WBC, white-cell count.

### 3.3 Univariate analysis of radiographic and ultrasound signs

Of the eight radiographic signs, portal venous gas, pneumatosis intestinalis, and pneumoperitoneum were significantly associated with surgery (all *P* < 0.001), and a rigid bowel course was borderline (*P* = 0.059). Of the eight ultrasound signs, bowel-wall thickening, pneumatosis intestinalis, portal venous gas, peritoneal effusion, and complex peritoneal effusion were all significantly associated with surgery (all *P* < 0.001). Complex peritoneal effusion was found in 27.3% of the surgical group but in only 0.5% of the conservative group [odds ratio (OR) = 78.56, 95% CI 18.13–340.43], making it the single most discriminating sign on univariate analysis (Table 3).

**Table 3:** Univariate analysis of radiographic and ultrasound signs.

Imaging sign	Surgical (*n* = 88, %)	Conservative (*n* = 421, %)	OR (95% CI)	*P*
Radiographic signs				
Intestinal gas	86 (97.7)	389 (92.4)	3.54 (0.83–15.04)	0.097
Rigid bowel course	20 (22.7)	59 (14.0)	1.80 (1.02–3.19)	0.059
Widened interloop spacing	42 (47.7)	177 (42.0)	1.26 (0.79–2.00)	0.389
Fixed bowel loop	0 (0.0)	2 (0.5)	–	1.000
Equivocal intramural gas	16 (18.2)	56 (13.3)	1.45 (0.79–2.67)	0.305
Pneumatosis intestinalis	13 (14.8)	17 (4.0)	4.12 (1.92–8.83)	<0.001
Portal venous gas	18 (20.5)	2 (0.5)	53.87 (12.23–237.28)	<0.001
Pneumoperitoneum	6 (6.8)	2 (0.5)	15.33 (3.04–77.29)	<0.001
Ultrasound signs				
Intestinal gas	86 (97.7)	408 (96.9)	1.37 (0.30–6.18)	1.000
Bowel-wall thickening	34 (38.6)	58 (13.8)	3.94 (2.36–6.57)	<0.001
Bowel-wall ischaemia	0 (0.0)	0 (0.0)	–	1.000
Equivocal intramural gas	2 (2.3)	6 (1.4)	1.61 (0.32–8.10)	0.632
Pneumatosis intestinalis	41 (46.6)	94 (22.3)	3.03 (1.88–4.89)	<0.001
Portal venous gas	24 (27.3)	39 (9.3)	3.67 (2.07–6.52)	<0.001
Peritoneal effusion	62 (70.5)	55 (13.1)	15.87 (9.26–27.19)	<0.001
Complex peritoneal effusion	24 (27.3)	2 (0.5)	78.56 (18.13–340.43)	<0.001

Compared by the *χ*^2^ test or Fisher’s exact test; OR, odds ratio; CI, confidence interval.

### 3.4 Multivariable regression and model comparison

**Figure 1:**
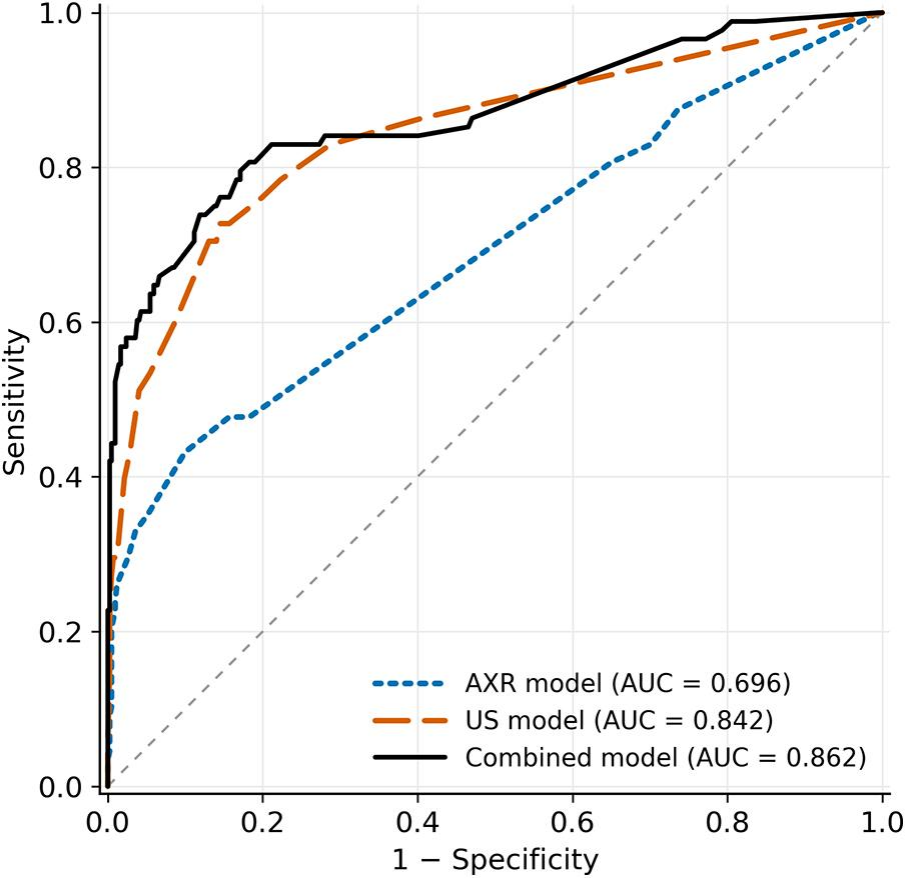
ROC curves of the radiographic, ultrasound, and combined models for predicting surgical intervention in NEC (*n* = 509). To ensure that the curves remain distinguishable in greyscale and for readers with colour-vision deficiency, the AXR model is drawn as a dotted line, the US model as a dashed line, and the combined model as a solid line; the grey dashed diagonal denotes chance.

Imaging variables with a univariate *P* < 0.10 were entered into the corresponding model. In the radiographic model, the independent predictors were portal venous gas (OR = 32.76, 95% CI 7.11–150.94, *P* < 0.001) and pneumoperitoneum (OR = 11.91, 95% CI 1.81–78.58, *P* = 0.010). In the ultrasound model, they were complex peritoneal effusion (OR = 16.94, 95% CI 3.65–78.76, *P* < 0.001), peritoneal effusion (OR = 7.53, 95% CI 4.04–14.03, *P* < 0.001), and portal venous gas (OR = 2.71, 95% CI 1.33–5.51, *P* = 0.006). The AUCs of the radiographic, ultrasound, and combined models were 0.696 (95% CI 0.638–0.754), 0.842 (95% CI 0.797–0.887), and 0.862 (95% CI 0.821–0.903), respectively (Table 4, Figure 1). Because radiographic portal venous gas and pneumoperitoneum had few events in the conservative group and produced wide 95% CIs under ordinary regression, we ran a Firth penalised model as a sensitivity analysis; the direction matched the main analysis: point estimates for the sparse variables shrank towards the median and their 95% CIs narrowed, while the significance of portal venous gas and pneumoperitoneum was unchanged.

**Table 4:** Discrimination and calibration of the three imaging models.

Model	AUC (95% CI)	Sensitivity (%)^a^	Specificity (%)^a^	PPV (%)	NPV (%)	HL *χ*^2^ (*P*)	Brier
Radiographic	0.696 (0.638–0.754)	27.3	98.3	77.4	86.6	1.17 (0.997)	0.116
Ultrasound	0.842 (0.797–0.887)	63.6	91.2	60.2	92.3	1.70 (0.989)	0.091
Combined	0.862 (0.821–0.903)	65.9	93.8	69.0	92.9	4.39 (0.820)	0.075

a At the cut-off of 0.3; PPV, positive predictive value; NPV, negative predictive value; HL, Hosmer–Lemeshow goodness-of-fit test.

**Figure 2:**
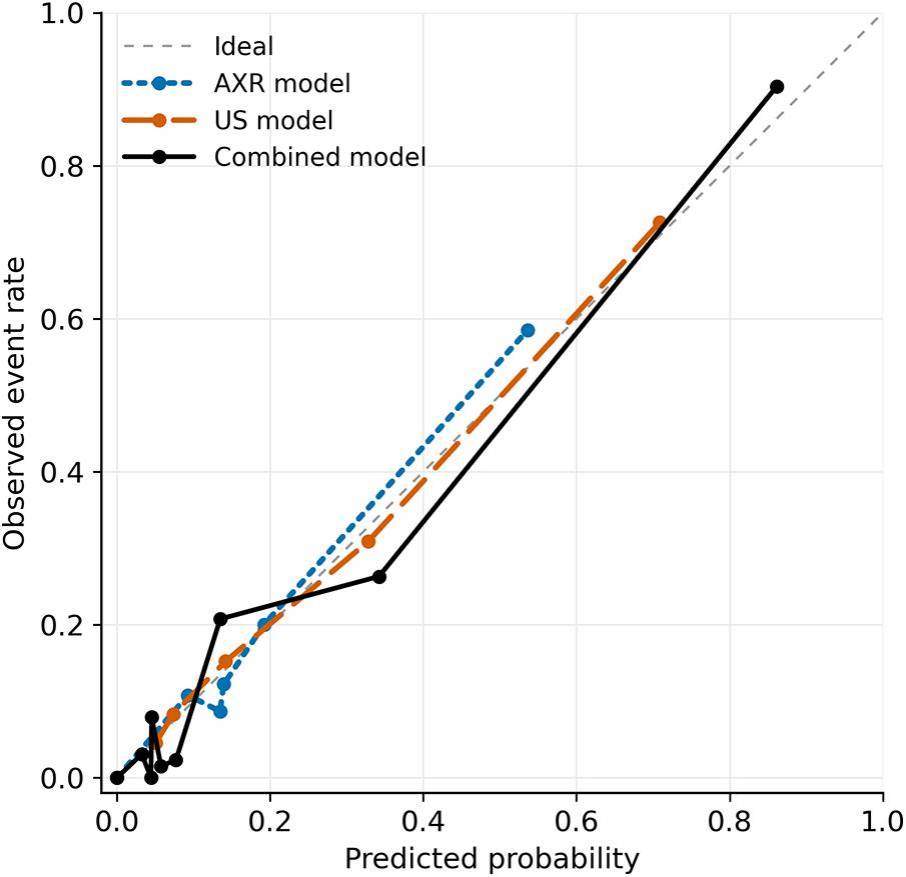
Calibration curves of the three models (grouped by deciles of predicted probability). The AXR model is shown as a dotted line, the US model as a dashed line, and the combined model as a solid line; the grey dashed line indicates ideal calibration.

On DeLong testing, both the ultrasound and combined models had higher AUCs than the radiographic model, with an *Δ*AUC of 0.147 (95% CI 0.075–0.218, *Z* = 4.03) and 0.167 (95% CI 0.104–0.229, *Z* = 5.24), both *P* < 0.001; the combined model exceeded the ultrasound model by only 0.020 (95% CI −0.001–0.041, *Z* = 1.83, *P* = 0.067), which was not significant. For reclassification, the combined model yielded an NRI of 1.074 and an IDI of 0.285 against the radiographic model and 0.823 and 0.106 against the ultrasound model, all statistically significant (*P* < 0.001). The HL *χ*^2^ values of the three models were 1.17, 1.70, and 4.39 (all *P* > 0.05), and the Brier scores were 0.116, 0.091, and 0.075; all were well calibrated, with the combined model coming closest to the observed event rate (Figure 2).

**Figure 3:**
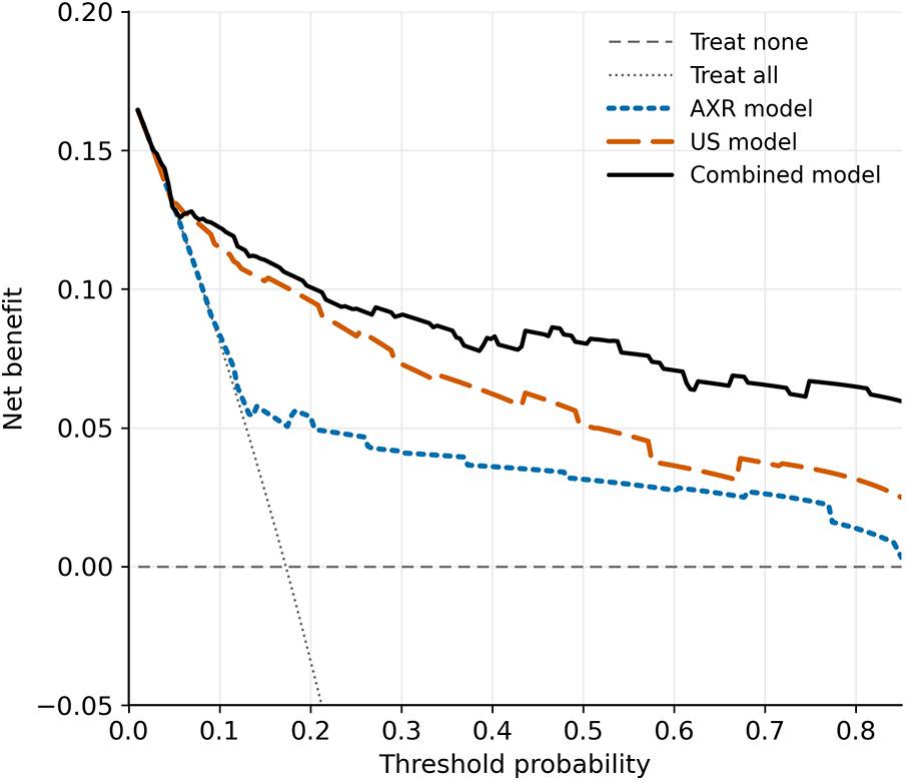
Net benefit of the three models on decision-curve analysis. The AXR model is shown as a dotted line, the US model, as a dashed line, and the combined model as a solid line.

At a cut-off of 0.3 (maximum Youden index), the sensitivities of the radiographic, ultrasound, and combined models were 27.3%, 63.6%, and 65.9%, the specificities 98.3%, 91.2%, and 93.8%, and the negative predictive values 86.6%, 92.3%, and 92.9%. A decision-curve analysis showed that across a wide threshold range of 0.05–0.6, the ultrasound and combined models had a greater net benefit than the radiographic model (Figure 3).

To address the selection bias inherent in the complete-case analysis, neonates diagnosed with NEC during the same period but without bedside ultrasonography were included, their ultrasound signs were imputed by multiple imputation with chained equations (*m* = 20), and the models were refitted. The AUCs of the ultrasound and combined models kept the same direction as the main analysis but fell in absolute terms, suggesting that the complete-case analysis may overestimate the performance of ultrasonography.

### 3.5 Subgroup analysis by gestational age

**Figure 4:**
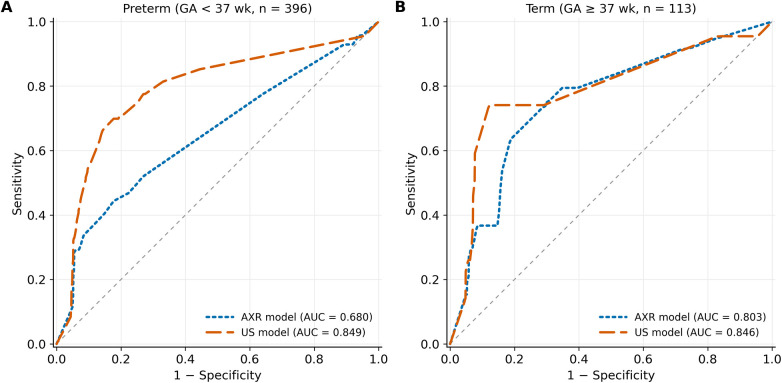
ROC comparison stratified by gestational age **(A)** preterm; and **(B)** term. In each panel, the AXR model is drawn as a dotted line and the US model as a dashed line; the grey dashed diagonal denotes chance.

We further stratified by gestational age. In the preterm subgroup (gestational age <37 weeks, *n* = 396, 71 operated), the radiographic and ultrasound models had AUCs of 0.680 and 0.849; in the term subgroup (*n* = 113, 17 operated), 0.803 and 0.846. Ultrasonography was no worse than radiography in either subgroup, and its advantage over radiography was more pronounced in preterm infants (Figure 4).

## 4 Discussion

The indication for surgery in NEC has long rested mainly on pneumoperitoneum seen on plain radiography; yet by the time pneumoperitoneum appears, the bowel is usually already necrotic through its full thickness, and intervention comes relatively late (3, 13). The 2025 ERNICA guideline similarly cautioned against relying on any single radiographic sign as an absolute surgical indication, recommending that imaging findings be weighed together with the clinical course (5). In a cohort of 509 neonates who had both abdominal radiography and bedside ultrasonography, we compared the two modalities directly: the ultrasound model discriminated better (AUC = 0.842) than the radiographic model (AUC = 0.696), and combining the two raised the AUC to 0.862 without loss of calibration. The combined model improved the NRI and IDI over the radiographic model, but its gain in the AUC over ultrasonography alone was not significant (*Δ*AUC = 0.020, *P* = 0.067), with the added value lying mainly in the reclassification of borderline cases.

The discriminative advantage of ultrasonography is largely attributable to its assessment of free peritoneal fluid. Complex peritoneal effusion was found in 27.3% of the surgical group but in only 0.5% of the conservative group, with a univariate OR of 78.56, the strongest of all imaging signs. This accords with the systematic review and meta-analysis by Cuna et al. (14), the review by May et al. (8), and the early controlled study by Muchantef et al. (15): turbid, septated, or loculated complex effusion tends to indicate local perforation or severe bowel necrosis, whereas simple anechoic effusion bears no clear relation to poor outcome, which is why we recorded “peritoneal effusion” and “complex peritoneal effusion” separately. Unlike the meta-analysis by Cuna et al. (14), in which ultrasound portal venous gas (pooled OR 3.0) and pneumatosis intestinalis (pooled OR 2.1) were not significantly linked to surgical outcome, ultrasound portal venous gas retained independent predictive value in our cohort (OR = 2.71, *P* = 0.006); the difference may relate to our stricter reading of the phase and duration of gas-bubble movement and also reflects how the degree of standardisation of the reading protocol affects the imaging–outcome link (14). Bowel-wall thickening (>2.7 mm, against the upper normal limit reported by Faingold et al. (12) and portal venous gas can both be assessed dynamically and repeatedly at the cot side without radiation exposure, which suits very-low-birth-weight infants particularly well (16).

Radiography cannot be wholly replaced by ultrasonography. On multivariable analysis, radiographic portal venous gas and pneumoperitoneum retained high independent ORs (32.76 and 11.91). However, adding radiography to ultrasonography did not significantly raise the AUC above that of ultrasonography alone (*Δ*AUC = 0.020, *P* = 0.067), suggesting that much of the information carried by these radiographic signs is already captured by ultrasonography. For fixed bowel loops, the overall course of the bowel, and the distribution of gastrointestinal gas, radiography still offers what ultrasonography cannot easily replace, and its reading is more standardised. Earlier studies are consistent with this: Priyadarshi et al. (17) showed in a prospective two-centre cohort of 67 NEC cases that radiography plus bedside ultrasonography improved surgical risk stratification over radiography alone (OR 6.96); Lazow et al. (18) built a multivariable score from radiography and ultrasonography with an AUC of 0.937; and Chen et al. (19) reported in a Chinese cohort of 164 cases that the ultrasound model had a higher AUROC (0.755) than the radiographic model (0.693). In a larger single-centre cohort, our study used the NRI and IDI to quantify the added value of combining the two, in keeping with these reports. In practice, bedside ultrasonography can be added to routine radiography in suspected or progressing NEC; when a neonate deteriorates rapidly but radiography does not yet show an absolute indication for surgery, complex peritoneal effusion on ultrasonography may serve as a reference for moving the timing of intervention forward (11, 19).

A stratified analysis showed that the advantage of ultrasonography over radiography was more pronounced in preterm infants (AUC 0.849 vs. 0.680), possibly because the irregular distribution of bowel gas in preterm infants makes radiographs harder to read; the radiographic model had a higher AUC in the term subgroup (0.803). The term subgroup, however, had only 17 surgical events and a low event-to-variable ratio, and therefore, its multivariable estimates are of limited stability and the finding should be read as a descriptive hypothesis awaiting confirmation. To address the question whether radiography retains more independent predictive information in term NEC, a larger sample is required.

This study has several limitations. The main one is selection bias: bedside ultrasonography is not a routine first-line examination but is usually added once clinicians judge the disease to be non-responsive to antibiotics and conservative management, and therefore, neonates with both radiography and ultrasonography tend to be sicker and at higher surgical risk. To gauge this, we refitted the models after multiple imputation with chained equations (*m* = 20) for neonates diagnosed with NEC over the same period but without ultrasonography; the AUCs of the ultrasound and combined models kept the same direction but fell in absolute terms, which suggests that the complete-case analysis may overestimate the performance of ultrasonography. In addition, signs such as radiographic pneumoperitoneum were rare in the conservative group and produced wide confidence intervals; although these were corrected with Firth’s penalised regression, the ORs spanning several orders of magnitude should still be read with caution. Imaging signs were taken from the original radiology and ultrasound reports and PACS records, and therefore, inter-reader subjectivity cannot be fully removed, and binary recording can only partly offset it; this will need confirmation in prospective studies with a standardised reading protocol. Because we used the original single clinical reports, each issued by one senior radiologist or sonologist, the images were not re-read independently in duplicate and inter-observer agreement (*κ*) could not be quantified. In addition, the models were developed and assessed in a single cohort using bootstrap-based internal estimation only; in keeping with the TRIPOD statement, the lack of a separate temporal or external validation cohort is a limitation, and the apparent performance reported here may be optimistic. The term subgroup had only 17 surgical events, and therefore, its conclusions are of limited stability; the study also did not include semi-quantitative bowel-wall perfusion scoring, contrast-enhanced ultrasonography, or other parameters. Multicentre prospective studies with standardised reading, combined with AI-based quantitative analysis, are the direction for future work (20).

For neonates with diagnosed or suspected NEC, bedside ultrasonography discriminates the need for surgical intervention better overall than abdominal radiography; combining the two does not further raise the AUC over ultrasonography alone but adds information at the level of reclassification. NICUs may consider incorporating bedside ultrasonography into the standard NEC imaging pathway as a complement to abdominal radiography, to help identify neonates needing surgery earlier and improve their outcome; a standardised adjunct-ultrasound protocol applied at the time of NEC diagnosis has recently been shown to be feasible and to detect abdominal pathology missed by radiography (21, 22). This is single-centre retrospective evidence, and the conclusions need confirmation in multicentre prospective studies.

## Data Availability

The original contributions presented in the study are included in the article/Supplementary Material, and further inquiries can be directed to the corresponding author.
